# Improving CLIP-seq data analysis by incorporating transcript information

**DOI:** 10.1186/s12864-020-07297-0

**Published:** 2020-12-17

**Authors:** Michael Uhl, Van Dinh Tran, Rolf Backofen

**Affiliations:** 1 Bioinformatics Group, Department of Computer Science, University of Freiburg, Georges-Köhler-Allee 106, Freiburg, 79110 Germany; 2 Signalling Research Centres BIOSS and CIBSS, University of Freiburg, Schaenzlestr. 18, Freiburg, 79104 Germany

**Keywords:** CLIP-seq, eCLIP, Peak calling, RBP binding site prediction

## Abstract

**Background:**

Current peak callers for identifying RNA-binding protein (RBP) binding sites from CLIP-seq data take into account genomic read profiles, but they ignore the underlying transcript information, that is information regarding splicing events. So far, there are no studies available that closer observe this issue.

**Results:**

Here we show that current peak callers are susceptible to false peak calling near exon borders. We quantify its extent in publicly available datasets, which turns out to be substantial. By providing a tool called CLIPcontext for automatic transcript and genomic context sequence extraction, we further demonstrate that context choice affects the performances of RBP binding site prediction tools. Moreover, we show that known motifs of exon-binding RBPs are often enriched in transcript context sites, which should enable the recovery of more authentic binding sites. Finally, we discuss possible strategies on how to integrate transcript information into future workflows.

**Conclusions:**

Our results demonstrate the importance of incorporating transcript information in CLIP-seq data analysis. Taking advantage of the underlying transcript information should therefore become an integral part of future peak calling and downstream analysis tools.

**Supplementary Information:**

The online version contains supplementary material available at (doi:10.1186/s12864-020-07297-0).

## Background

Over the last decade, CLIP-seq (cross-linking and immunoprecipitation followed by next generation sequencing) [1] has become the state-of-the-art procedure to experimentally determine the precise transcriptome-wide binding locations of RNA-binding proteins (RBPs). Many variants have been introduced, out of which PAR-CLIP [2], iCLIP [3], and eCLIP [4] are currently the most widely used. Regardless of the variant, CLIP-seq is usually applied in vivo to a specific RBP, producing a library of reads bound by the RBP. Identification of binding sites is subsequently achieved by mapping the reads back to the corresponding reference genome and running a so called peak caller tool on the read profiles. A number of popular peak callers have emerged over the years, such as Piranha [5], CLIPper [6], PEAKachu [7], and PureCLIP [8].

While there exist various protocol-specific as well as more generic peak callers [9], none of the current tools takes into account the transcript information underlying the mapped reads. Instead, they extract binding regions directly from the genomic read profiles. This can be acceptable if the studied RBP binds intronic sequences or in general unspliced RNAs. However, if the RBP is actually predominantly binding to spliced RNAs, which should be true for most cytoplasmically active RBPs, ignoring transcript information potentially leads to false peak calling and the inclusion of non-authentic sequence context. This in turn can compromise the results of downstream analysis tools like motif finders or binding site predictors, which usually take the genomic sequence context for extending the binding sites as well.

Here we show that current peak callers indeed have problems with correctly defining binding sites for RBPs binding predominantly to exonic regions. We further look at publicly available eCLIP datasets with binding sites identified by CLIPper and present comprehensive statistics regarding exonic binding frequencies. Focusing specifically on sites near exon borders, we report the extent of sites mostly affected by context sequence selection and false peak calling. To compare different sequence contexts, we implemented a tool called CLIPcontext. CLIPcontext automatically extracts the transcript and genomic context for a given set of transcript or genomic sites, and also offers other useful functions such as identifying sites at exon borders or motif search. We then trained three different binding site prediction tools on sites near exon borders, and demonstrate that sequence context choice can have a large impact on predictive performance. Moreover, we show for a selection of predominantly exon-binding RBPs that known motifs are enriched in transcript context sequences, enabling the identification of more authentic binding sites. In the end, we discuss possible ways on how to integrate transcript information in order to improve CLIP-seq data analysis workflows.

## Results and discussion

### Ignoring transcript information compromises peak calling

**Fig. 1:**
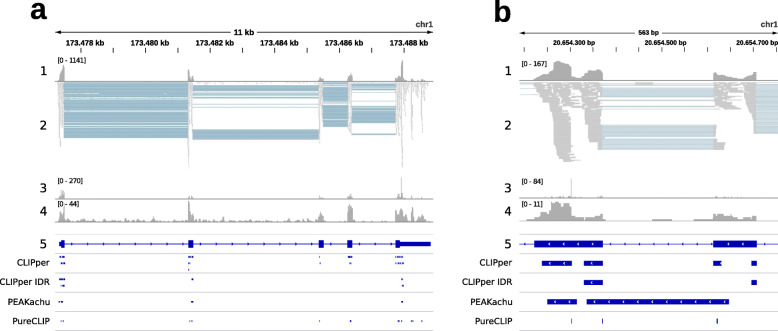
IGV snapshot of two genomic regions with mapped YBX3 K562 eCLIP data. 1: read profile (coverage), 2: read alignments, 3: crosslink positions profile, 4: input control profile, 5: gene annotations (thick blue regions are exons, thin blue regions introns), CLIPper / CLIPper IDR: CLIPper replicate 1 and IDR peaks, PEAKachu: PEAKachu peaks, PureCLIP: PureCLIP peaks (nearby crosslink positions merged). For clarity only gene strand reads from replicate 1 are displayed. **a***PRDX6* whole gene region (length 11 kb, maximum read coverage 1141). **b***DDOST* gene exons 6 and 7 region (length 563 bp, maximum read coverage 167)

To illustrate the issues current peak callers have with predominantly exon-binding RBPs, we chose one out of many eCLIP RBP cell type combinations (YBX3 K562) with large amounts of exonic binding regions (see Table S1 for eCLIP overlap statistics). In this paper, we call or count peak regions as overlapping or exon binding if they have an overlap of ≥90% with exonic regions. 84.6% of YBX3 K562 merged peak sites overlap with exonic regions, out of which 51.0% are ≤50 nt away from exon borders. Figure 1 shows the YBX3 K562 genomic reads profile visualized via IGV (Integrative Genomics Viewer) [10] over two genomic regions, with added peak regions from CLIPper, CLIPper IDR, PEAKachu, and PureCLIP (see Methods section “Peak caller setup”). Figure 1a depicts a genomic region of 11 kb, containing the *PRDX6* gene. We can see that the read alignments clearly follow the exon annotations: most reads map to exons, including many intron-spanning ones (blue-gray lines), while only few reads map to introns. Not surprisingly, all three peak callers only report exonic peaks, often close or directly at exon borders. Given the alignment information, extending these peak regions with genomic context, as usually done prior to further analysis, is not correct. Instead, the transcript context of the spliced RNA should be used, which is where the actual RBP binding occurs. Zooming in on the matter, Fig. 1b shows a genomic region of 563 bp, comprising exon 6 and 7 of the *DDOST* gene. Again the mapped reads strongly suggest a spliced RNA context, given the many intron-spanning reads and almost no intron coverage. Keeping the intron therefore leads to an artificial split-up of peak regions spanning the exon border. Unaware of the split, peak callers might consequently call two peaks, whereas they should have treated the split peaks as one contiguous region.

In the Fig. 1b example, both CLIPper and PureCLIP call peaks at adjacent exon borders, while PEAKachu even calls a single peak over the entire intron. In general, PEAKachu and CLIPper define peak regions by fitting functions (Gaussian density versus splines) on the mapped reads. More precisely, CLIPper fits splines on the genomic read coverage profile counting each base of a read once, while PEAKachu replaces each read with a Gaussian, using the genomic mean of read start and end as the center of the Gaussian. Both methods thus have problems with split reads, leading to PEAKachu calling peaks over introns in the presence of intron-spanning reads, and CLIPper calling peaks at exon ends with shared read coverage. Using more robust peaks (like CLIPper IDR) is the recommended way to obtain high-confidence binding sites, but it does not solve the underlying issue (see also Fig. 3). In contrast, PureCLIP uses read starts to identify crosslink sites, which later can be merged into peak regions. This circumvents the described problems, as each read is considered only once at one genomic position. For example, Fig. 1b shows a peak called by CLIPper and CLIPper IDR at the start of exon 6 (downstream exon). But since there are no read starts (i.e., crosslink sites) present, PureCLIP does not call a peak here. On the other hand, it still can be fooled since intron-spanning reads are treated no different to contiguously aligned reads. For the YBX3 dataset and with default settings, PEAKachu tends to call broader peaks than CLIPper, while PureCLIP peaks are much shorter (see Table S2 for peak statistics).

### Exon binding is substantial in public CLIP-seq data

**Fig. 2:**
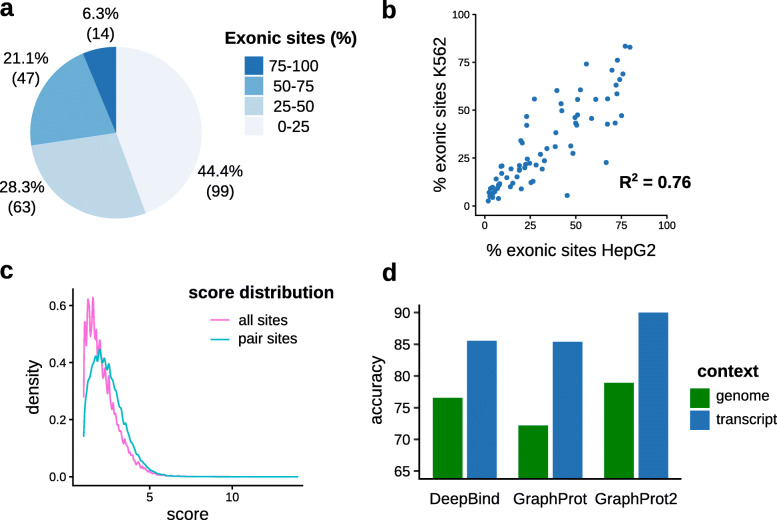
Exon binding statistics of eCLIP datasets and prediction results for different sequence contexts. **a** Distribution of exonic site ratios for 223 eCLIP datasets over four percentage ranges. For each range, the percentage (number) of sets with ratios falling into this range is given. **b** Correlation plot of exonic site ratios for RBPs present in two cell lines (HepG2 and K562). **c** Site score distributions for all exonic sites and exonic sites that form pairs by being located at adjacent exon borders. *log*_2_ fold change values of the sites determined by CLIPper were taken as site scores. Only pair sites with a distance of <10 nt to exon borders were considered. **d** Average classification accuracies over 6 eCLIP datasets for 3 RBP binding site prediction methods, comparing genome and transcript context

To quantify the extent of exon and near exon border binding in eCLIP data, CLIPper peak regions from 223 eCLIP datasets were overlapped with exon regions featuring strong experimental evidence (see Methods section “Data preparation and exon overlap statistics”). As shown in Fig. 2a, 61 datasets (27.4%) feature ≥50% exonic sites, with 14 datasets (6.3%) even reaching ≥75% (see Table S1 for full statistics on each dataset). Table S1 also lists the ratios of sites near exon borders and pair sites, i.e., two sites located at adjacent exon borders. Looking closer at the 61 datasets, 63.3% of exonic sites lie within ≤50 nt to exon borders, and 20.7% form pairs (<10 nt distance of site ends to exon borders required for both sites of the pair). We thus have a substantial amount of sites susceptible to split peak calling and false sequence context choice. Since the selection procedure for splice isoforms (i.e., their exon regions) was quite strict, the actual percentages should be even higher. As the data features experiments from K562 and HepG2 cell lines, we also looked at the correlation of percentages for RBPs with experiments in both lines. Figure 2b shows the correlation plot of exon site ratios, resulting in an *R*^2^ score of 0.76. This suggests a general agreement in the amount of exon binding across cell lines. On the other hand, it also shows that classifying RBPs into spliced or unspliced binding oversimplifies actual binding patterns. Instead, the correct site context needs to be determined directly from the mapped data. One might wonder whether potentially problematic pair sites could easily be filtered out based on their assigned scores (i.e., *log*_2_ fold changes) prior to data analysis. As shown in Fig. 2c, this is not the case, with an average score of 2.47 for pair sites and 2.17 for all exonic sites.

**Fig. 3:**
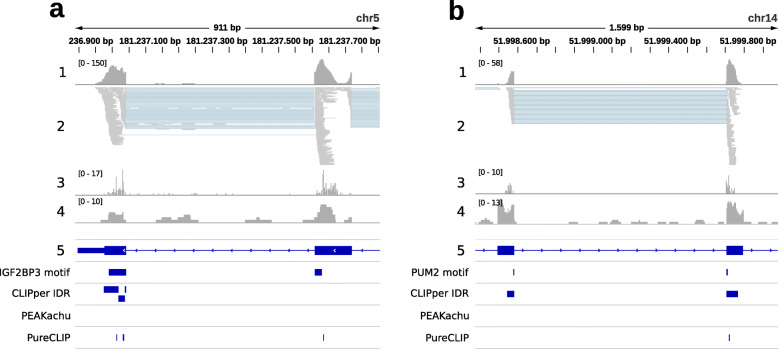
IGV snapshot of two genomic regions with mapped IGF2BP3 K562 and PUM2 K562 eCLIP data. 1: read profile (coverage), 2: read alignments, 3: crosslink positions profile, 4: input control profile, 5: gene annotations (thick blue regions are exons, thin blue regions introns), IGF2BP3 / PUM2 motif: RBP motifs mapped with CLIPcontext, CLIPper IDR: CLIPper IDR peaks, PEAKachu: PEAKachu peaks, PureCLIP: PureCLIP peaks (nearby crosslink positions merged). For clarity only gene strand reads from replicate 1 are displayed. **a***RACK1* gene exons 7 and 8 region (length 911 bp, maximum read coverage 150) with split IGF2BP3 motif hit. **b***RTRAF* gene exons 4 and 5 region (length 1.599 bp, maximum read coverage 58) with split PUM2 motif hit

### Sequence context influences binding site prediction performances

Based on the considerable amount of sites near exon borders, we further investigated whether different sequence contexts could also influence the performances of binding site prediction tools. For this we constructed different context datasets for 6 RBPs, by focusing on RBPs with high amounts of exonic sites (≥80%) and choosing 5 RBPs randomly within this range (see “Methods” sections). Briefly, we kept only sites ≤10 nt from exon borders and extended the centered sites 80 nt up- and downstream with both genomic and transcript context (total length 161 nt, see Table S3 for dataset details). Note that this also includes sites at transcript ends, where full extension is only possible in the genomic context case. To assess any effects, three different prediction tools (DeepBind [11], GraphProt [12], and GraphProt2 [13]) were run on both context sets, using 10-fold cross validation and no additional features (i.e., only sequence information). Figure 2d shows the performance results as average accuracies over the 6 datasets, for both genomic and transcript context sets (see Table S4 for detailed results). As we can see, using the more authentic transcript context considerably improves accuracies for all three tools, showcasing that context sequence choice can have a large influence on predictive performance and thus on what is learned. One could argue that including large amounts of context sequence bears the risk of learning binding site-unspecific patterns. We acknowledge that this can influence predictions. Some bias from the negative set is also possible, although we tried to minimize this by random sampling from the whole gene sequence and no overlap with positive sites. On the other hand, intronic context near exon borders also harbors various recognizable regions, like the polypyrimidine tract, or splice donor and acceptor sites, which can lead to wrong conclusions for spliced RNA binding RBPs. Moreover, learning the transcript context for RBPs binding to spliced RNA can also be advantageous, especially when predicting on gene sequences that contain introns.

To check whether the trained models learned any RBP specific binding information or rather generic context features, we generated GraphProt sequence logos for each RBP-context combination (see Figure S1). Sequence logos are generated from the top 200 scoring sites (taking the highest scoring 8-mer sequence for each site) of each positive training set, therefore providing a visualization aid of what sequence information the model regards as most important. Comparing the generated sequence logos with known RBP binding preferences obtained from the ATtRACT database [14], we can see a general agreement (more or less pronounced depending on the RBP). For example, the Pumilio Response Element (PRE) of PUM2 (UGUANAUA) clearly shows up for both context sets, as well as the preference for CA-rich elements for IGF2BP1 and YBX3 or GA-rich elements for SRSF1. FMR1 and FXR2 are less distinguishable, although both RBPs are closely related and thus also might have common targets. This indicates that the models do not primarily pick up generic context information, but instead are capable of prioritizing RBP specific binding sites, independent of the context. Nevertheless, since we included a large amount of context (sequence lengths 161 nt), the context is expected to contribute to the increased performances for the transcript context sets. As discussed, this can be, depending on the prediction task, beneficial, as it can offer new insights into what other elements tend to be associated with core binding elements. In addition, choosing a more authentic context could also help to improve RNA secondary structure predictions, which often include hundreds of nucleotides of context.

### Known motifs are enriched in transcript context

To check whether known binding motifs are more frequent in eCLIP sites with added transcript context compared to the respective sites with genomic context, we collected 28 motifs from 9 RBPs known to bind predominantly to spliced or exonic RNA (FMR1, FXR1, FXR2, IGF2BP1-3, PUM2, SRSF1, and YBX3) [15–20]. Since we could not find reported human motifs for YBX3, we used the corresponding mouse motif [21], as well as two human motifs from YBX1 and YBX2. We then took the CLIPper IDR peak regions (high-confidence reproducible peaks between replicates) of the respective eCLIP datasets, and used CLIPcontext to select sites near exon borders and to look for motifs in both genomic and transcript context sites. As shown in Table S5, there are 23 motifs that have >10 hits in both genomic and transcript context sites (counting hits at same genomic or transcript positions only once). Out of these 23, 20 are 10 - 57% more frequent in transcript context sites, while the remaining three change by 2.8%, -2.3%, and -2.4%. The other five motifs with less than 10 hits are all enriched by 35% up to 709% (ratios calculated with counts normalized by total context set length).

On the one hand, when taking the transcript context, we expect higher frequencies for motifs that are commonly found in exonic regions. On the other hand, well-defined motifs like the PUM2 PRE (107 vs. 89 hits, 27.5%) or the extended compound motif for IGF2BP3 (7 vs. 1 hit, 709%) also show increased frequencies, indicating that more authentic binding sites are recovered by using the transcript context. To illustrate this (Fig. 3), we chose two example regions that contain IDR peaks as well as known binding motifs mapped by CLIPcontext of IGF2BP3 (the mentioned recently published compound motif GGC-N _15−25_-CA-N _7−20_-CA-N _15−25_-GGC-N _2−8_-[CA]_4_) and PUM2 (the mentioned PRE UGUANAUA). As shown, the motifs are even split in these examples by the exon border, and the read profile accordingly suggests one split peak, although multiple CLIPper IDR peaks are reported, either in one of the two exons (IGF2BP3), or one at each adjacent exon end (PUM2). Naturally, we would expect the influence of context choice on recovering complete binding sites to be higher for multi-domain RBPs like IGF2BP1-3, which prefer to bind to several disconnected elements with long stretches of variable length in between. Since most RBPs in fact contain multiple RNA-binding domains and systematic studies on their combinatorial RNA recognition are still scarce [20], identifying the correct context in CLIP-seq studies could further help to uncover their combinatorial binding modes.

### Strategies to improve CLIP-seq data analysis workflows

In this study we used CLIPcontext to extract the transcript context of genomic sites from a set of well annotated splice isoforms, completely ignoring the context information given in the eCLIP data. This is of course far from optimal, and future workflows should implement a more sophisticated, data-driven way to incorporate transcript information, in order to identify the most likely context and therefore increase the accuracy of peak calling and downstream processes. In this regard, one major factor will be the ability to correctly identify exon regions and their corresponding isoforms in a given sample, or at least the correct site neighborhood for accurate context extraction. The presence and quantity of split reads at exon borders therefore marks an important feature to decide which context is appropriate. Unfortunately, reference annotations often lag behind and do not cover the present transcript diversity [22], which is why de novo transcriptome assemblies from RNA-seq data, e.g. by tools like Ryūtō [23], might be an interesting alternative to isoform detection or mapping approaches that rely on reference annotations. Since all these tools were developed for RNA-seq data, it will also be interesting to see whether it is possible to adapt them to work directly with CLIP-seq data, omitting the need to conduct additional RNA-seq experiments.

In any case, context selection should ideally be done on site level, as RBPs often have several biological roles and can bind to both contexts, depending on subcellular location and the time point in the RNA life cycle. In this regard, applying CLIP-seq to different subcellular fractions might be a way to further dissect binding events, as already done for some multi-function SR proteins [24]. In the presence of several likely contexts (i.e., for alternative splice isoforms), it is possible to keep all events if the goal is to learn general binding characteristics. This is because binding site prediction tools are typically robust when it comes to noisy data, as long as the principal binding preferences are still present in sufficient quantities. However, if the focus lies on specifically studying these events, it would be most convenient to label and output them separately.

An alternative approach could be to adapt or fine-tune peak calling based on specific features of the dataset at hand. These features could be learned from publically available CLIP-seq datasets, ideally produced with the same protocol (including read mapping), and possibly also the same cell type or condition. For example, dataset properties could be extracted and used as features, like exon-intron read distributions for typical exon-, intron-, or mixed context binding RBPs, either at defined genomic locations or over the whole genome. Additional labeled test data (either derived from CLIP-seq data or artificially constructed) could then be used to evaluate what features or strategies work best.

## Conclusions

In this paper we raised the issue of ignoring transcript information in the process of peak calling and beyond. We showed that current peak callers by design are prone to false peak calling near exon borders, and that peak regions near exon borders are frequent in publicly available datasets. We also saw that sequence context choice has a profound effect on predicting sites near exon borders. Moreover, motif analysis confirmed that choosing the transcript context enriches for known RBP binding motifs, leading to the recovery of more authentic binding sites. Finally, we discussed ways on how to improve CLIP-seq analysis workflows in order to identify the correct site context.

Taken together, incorporating transcript information leads to more authentic results and thus should become an integral feature of future peak calling and downstream analysis methods.

## Methods

### Data preparation and exon overlap statistics

eCLIP datasets out of two cell lines (HepG2, K562) were downloaded from the ENCODE project website [25] (https://www.encodeproject.org, November 2018 release). Altogether the data covers 150 RBPs, divided into 103 HepG2 and 120 K562 sets, resulting in 223 datasets. We directly used the genomic binding regions (genome assembly GRCh38) determined by CLIPper, available in BED format for each replicate (2 replicates per dataset). For each RBP cell type combination, replicate binding sites were merged by keeping only the sites with the highest *log*_2_ fold change (LFC) in case of overlapping sites. After filtering sites by LFC ≥1, sites were overlapped with exon regions of the most prominent transcripts using intersectBed (bedtools 2.29.0 [26]) and a required exon overlap ≥90% for a region to be counted as exon overlapping. We defined the most prominent isoform of a gene based on the information Ensembl (Ensembl Genes 97, GRCh38.p12) provides for each transcript through hierarchical filtering: APPRIS annotation [27] (highest priority, labels principal1-5), and transcript support level (TSL, labels 1-5). We considered only genes with isoforms featuring these labels and transcripts that belong to the GENCODE basic gene set, resulting in 29,798 isoforms and 238,271 exon regions. Exon overlap statistics for the 223 datasets are stored in Table S1.

### Peak caller setup

To illustrate potential peak caller problems (Fig. 1), we chose an RBP cell type combination with a high amount of exonic peak regions (YBX3 K562, 84.6%), out of which 51.0% are close to exon borders (region ends ≤50 nt from exon borders, see Table S1 for statistics). To illustrate false peak calling at sites containing known motifs (Fig. 3), we chose the IGF2BP3 (HepG2) and PUM2 (K562) eCLIP sets. Mapped eCLIP reads in BAM format (replicate 1, size-matched input) and CLIPper peak regions (BED) for the three sets (ENCODE IDs ENCSR529FKI, ENCSR993OLA, ENCSR661ICQ) were obtained from the ENCODE website.

We collected peak regions identified by three peak callers: CLIPper, PEAKachu, and PureCLIP. For CLIPper, we took the peak regions called on replicate 1, filtered by a minimum LFC of 1. In addition, we also display the CLIPper IDR peaks (high-confidence peaks reproducible between replicates, Figs. 1 and 3). For PEAKachu and PureCLIP, we took the mapped reads (replicate 1, size-matched input), and used the R2 reads (second pair reads) as experiment and control libraries. PEAKachu was run on Galaxy [28] (https://usegalaxy.eu, Galaxy tool version 0.1.0.2) with default settings and a fold threshold of 2. PureCLIP (version 1.3.1) was installed locally and run with default parameters, setting −*dm* 8 for merging called crosslink sites into peak regions.

### Construction of sequence context sets

For comparing the effects of different sequence contexts on predictive performance, we chose 6 eCLIP sets from RBPs with documented binding preferences (IGF2BP1, FMR1, FXR2, PUM2, SRSF1, YBX3), which also feature relatively high percentages of exonic peak regions (from 40.23 to 84.06%, see Table S1). CLIPper replicate 1 peaks were obtained and filtered (maximum length of 80, minimum LFC of 3, maximum *p*-value of 0.01). We further selected all exonic sites within ≤10 nt of exon borders (clipcontext exb), and extracted their transcript and genomic context (clipcontext g2t), merging nearby sites (distance ≥10 nt) by selecting the site with the highest LFC, and extending sites to 161 nt length. To generate one negative set for both genome and transcript context sets, we used GraphProt2 (https://github.com/BackofenLab/GraphProt2) to randomly select genomic sites based on two criteria: 1) their location on genes covered by eCLIP peak regions and 2) no overlap with any eCLIP peak regions from the experiment. Sequence context set statistics are stored in Table S3.

### Tool setup for context predictions

Three RBP binding site prediction tools (DeepBind, GraphProt, and GraphProt2) were trained on the described context sets (see previous Methods section). DeepBind models were trained using the DeepRAM [29] framework, which includes hyperparameter optimization. GraphProt and GraphProt2 models were trained using default parameters (no hyperparameter optimization). All three methods used only sequence features for classification. The accuracy measure, i.e., the proportion of correctly classified instances, was used in combination with 10-fold cross validation to measure model performances over 6 datasets. Accuracies are reported in Table S4, together with standard deviations from cross validation (except for GraphProt, since it does not output single accuracies during cross validation). GraphProt sequence logos for the top 100 scoring sites of each dataset-context combination are shown in Table S5, together with a description of known binding preferences.

### Motif search

For the motif search, CLIPper IDR peaks for 9 RBPs were downloaded from ENCODE and filtered by a maximum length of 80. Sites near exon borders were selected and their transcript and genomic context was extracted as described in section “Construction of sequence context sets”. CLIPcontext (clipcontext mtf) was then used to obtain motif frequencies in the transcript and genomic context sets, as well as to map the PUM2 and IGF2BP3 motifs to the genome, to generate the split motif annotations seen in Fig. 3.

### CLIPcontext availability and documentation

CLIPcontext is available together with a comprehensive documentation on GitHub (https://github.com/BackofenLab/CLIPcontext), as well as on Bioconda (https://anaconda.org/bioconda/clipcontext). Besides mapping sites of interest in BED format (transcript or genomic coordinates) to a user-definable transcriptome or the genome, CLIPcontext also offers modes for the extraction of: sites near exon borders, a list of most prominent transcripts, intronic sites, or exon and intron regions for a given set of transcripts. Moreover, a motif search can be conducted on genomic and transcript regions (including split motif discovery) for comparative analysis.

## Supplementary Information

**Additional file 1** Table S1: Exon overlap statistics of ENCODE eCLIP datasets (.xlsx)

**Additional file 2** Supplementary tables S2-S4 and supplementary figure S1 (.pdf)

**Additional file 3** Table S5: Motif search results for 9 RBPs and 28 binding motifs collected from various sources (.xlsx)

## Data Availability

CLIPcontext is available on GitHub (https://github.com/BackofenLab/CLIPcontext) and Bioconda (https://anaconda.org/bioconda/clipcontext). Supplementary data is also stored in the GitHub repository.

## Abbreviations

CLIP-Seq: Cross-linking and immunoprecipitation followed by next generation sequencing
eCLIP: Enhanced CLIP
iCLIP: Individual-nucleotide CLIP
IGV: Integrative genomics viewer
PAR-CLIP: Photoactivatable-ribonucleoside-enhanced CLIP
RBP: RNA-binding protein
nt: Nucleotides
LFC: Log2 fold change
PRE: Pumilio response element
