# Supplementary material for: Improving CLIP-seq data analysis by incorporating transcript information
Source: BMC Genomics. 2020 Dec 17;21:894. doi: 10.1186/s12864-020-07297-0 (PMC7745353; doi:10.1186/s12864-020-07297-0)
Supplement: Supplementary file 2 — Additional file 2 Supplementary tables S2-S4 and supplementary figure S1 (.pdf) [file 12864_2020_7297_MOESM2_ESM.pdf]

# Improving CLIP-seq data analysis by incorporating transcript information

Supplementary Material

Michael Uhl, Van Dinh Tran, and Rolf Backofen

November 27, 2020

## Supplementary tables

Table 1: Exon overlap statistics of ENCODE eCLIP datasets (see Additional File 1 in .xlsx format).

Table 2: Peak length statistics for CLIPper (replicate 1), CLIPper IDR, PEAKachu, and PureCLIP on YBX3 K562 replicate 1 eCLIP data. Peaks were called as described in supplementary methods section "Peak caller setup". Introns for determining overlapping sites were selected based on the set of exons extracted, as described in methods section "Data preparation and exon overlap statistics". A site is counted as intron-spanning if it completely overlaps with an intronic region.

| Metric | CLIPper | CLIPper IDR | PEAKachu | PureCLIP |
|---|---|---|---|---|
| # sites | 132,842 | 17,982 | 11,537 | 54,308 |
| # sites > 500 nt | 0 | 0 | 471 | 0 |
| # intron-spanning sites | 4 | 2 | 1,096 | 0 |
| Minimum length | 1 | 1 | 18 | 1 |
| Maximum length | 263 | 217 | 22,875 | 25 |
| Mean length | 37.9 | 28.0 | 112.4 | 1.6 |
| Median length | 34 | 27 | 48 | 1 |
| 25th percentile | 19 | 13 | 42 | 1 |
| 75th percentile | 51 | 50 | 64 | 2 |

Table 3: Dataset statistics for the 6 eCLIP sets used for genomic and transcript context comparison. A minimum $log_2$ fold change (LFC) of 3 and a maximum p-value (PV) of 0.01 was used for filtering initial CLIPper replicate 1 peak sites. Moreover, only exonic sites (overlapping $\geq$ 90% with exons) near exon borders ($\leq$ 10 nt away) were selected. In case of overlapping sites ($\leq$ 10 nt distance), only the site with the highest LFC was kept. Positives: number of positive training instances. Negatives: number of negative training instances.

| RBP | Cell type | LFC | PV | Positives | Negatives |
|---|---|---|---|---|---|
| FMR1 | K562 | 3 | 0.01 | 2569 | 2569 |
| FXR2 | K562 | 3 | 0.01 | 3166 | 3166 |
| IGF2BP1 | K562 | 3 | 0.01 | 2199 | 2199 |
| PUM2 | K562 | 3 | 0.01 | 1136 | 1136 |
| SRSF1 | K562 | 3 | 0.01 | 1049 | 1049 |
| YBX3 | K562 | 3 | 0.01 | 4370 | 4370 |

Table 4: Performance results for 6 RBP eCLIP sets with genomic and transcript context. We report average accuracies obtained by 10-fold cross validation together with standard deviations (apart from GraphProt).

| Methods | RBP | Cell line | Genomic context | Transcript context |
|---|---|---|---|---|
| DeepBind | FMR1 | K562 | 80.63±1.58 | 88.22±1.99 |
| | FXR2 | K562 | 76.93±2.66 | 86.93±1.18 |
| | IGF2BP1 | K562 | 75.72±2.59 | 83.90±2.08 |
| | PUM2 | K562 | 70.05±2.94 | 80.69±2.31 |
| | SRSF1 | K562 | 79.39±4.64 | 85.98±3.07 |
| | YBX3 | K562 | 76.63±2.73 | 87.32±1.24 |
| GraphProt | FMR1 | K562 | 78.47 | 88.50 |
| | FXR2 | K562 | 75.71 | 86.73 |
| | IGF2BP1 | K562 | 66.24 | 84.18 |
| | PUM2 | K562 | 64.88 | 79.58 |
| | SRSF1 | K562 | 76.41 | 86.61 |
| | YBX3 | K562 | 71.63 | 86.61 |
| GraphProt2 | FMR1 | K562 | 82.66±1.68 | 92.23±1.11 |
| | FXR2 | K562 | 81.51±1.57 | 91.09±0.84 |
| | IGF2BP1 | K562 | 75.58±1.81 | 88.54±1.83 |
| | PUM2 | K562 | 73.86±1.95 | 86.27±2.76 |
| | SRSF1 | K562 | 80.93±3.01 | 91.09±2.54 |
| | YBX3 | K562 | 79.22±1.20 | 90.86±0.53 |

Table 5: Motif search results for 9 RBPs and 28 binding motifs collected from various sources (see Additional File 3 in .xlsx format).

## Supplementary figures

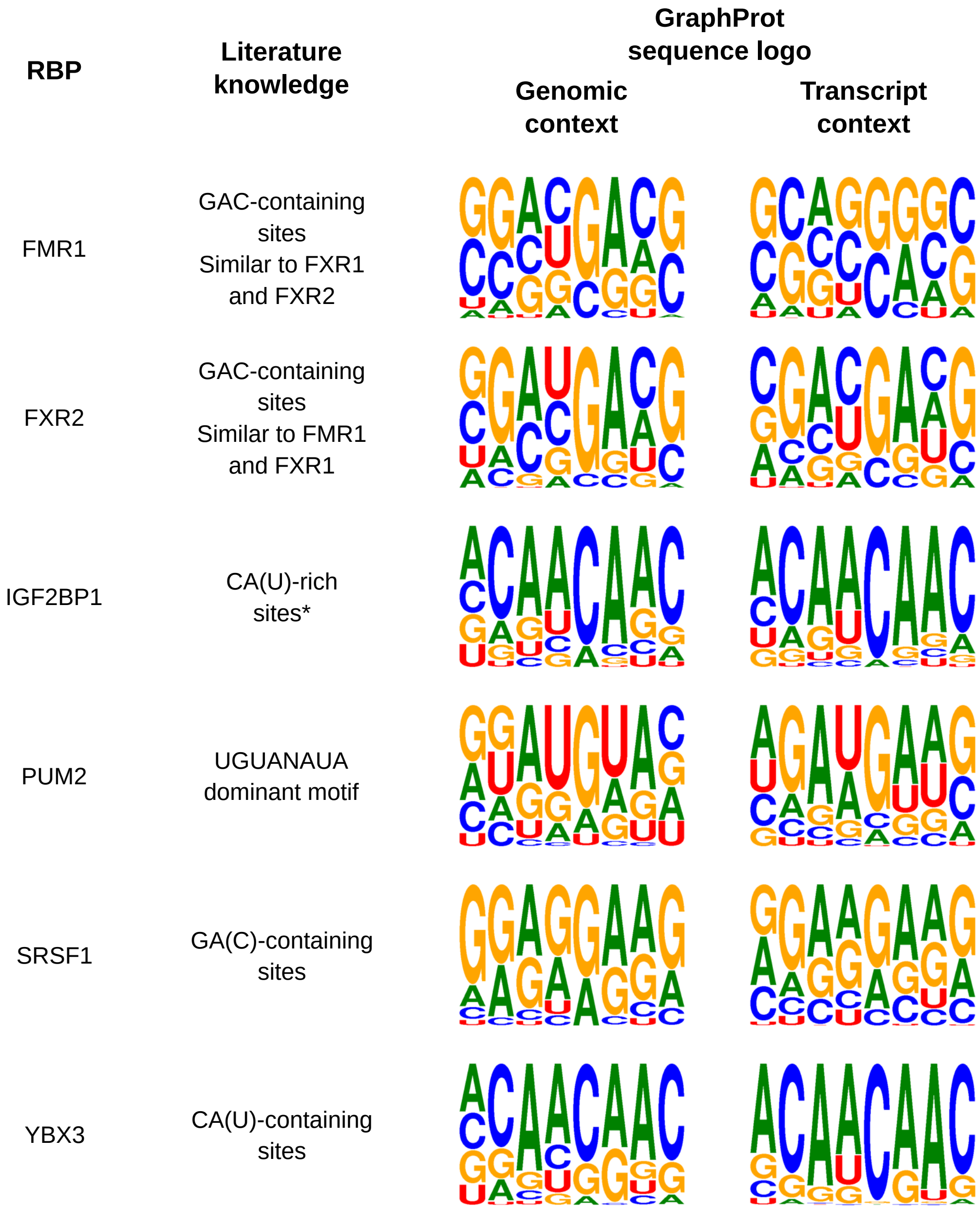


Figure 1: GraphProt sequence logos generated from models trained on the 6 eCLIP sets with genomic and transcript context (resulting in 12 models and 12 logos). Literature knowledge regarding RBP binding preferences was obtained from the ATtRACT database [1]. A logo is constructed for each RBP-context combination from the top 200 scoring sites (taking highest scoring 8-mer sequence for each site) of the positive set. *: note that IGF2BP1 binding sites are comprised of several parts, of which one dominant part are CA(U) rich sites.

.

## References

[1] Giudice, G., Sánchez-Cabo, F., Torroja, C., Lara-Pezzi, E.: ATtRACT - a database of RNA-binding proteins and associated motifs. Database **2016** (2016)
